# One size does not fit all in severe infection: obesity alters outcome, susceptibility, treatment, and inflammatory response

**DOI:** 10.1186/cc12794

**Published:** 2013-06-20

**Authors:** Petch Wacharasint, John H Boyd, James A Russell, Keith R Walley

**Affiliations:** 1 University of British Columbia, Critical Care Research Laboratories, Institute for Heart + Lung Health, St. Paul's Hospital, 1081 Burrard Street, Vancouver, BC, Canada V6Z 1Y6

## Abstract

**Introduction:**

Obesity is an increasingly common comorbidity in critically ill patients. Whether obesity alters sepsis outcome, susceptibility, treatment, and response is not completely understood.

**Methods:**

We conducted a retrospective analysis comparing three group of septic shock patients based on the intervals of actual body mass index (BMI) in patients enrolled in the VASST (Vasopressin and Septic Shock Trial) cohort. Primary outcome measurement was 28-day mortality. We tested for differences in patterns of infection by comparing the primary site of infection and organism. We also compared the treatments (fluids and vasopressors) and inflammatory response, measuring adipose tissue-related cytokine concentrations (interleukin [IL]-6, monocyte chemotactic protein [MCP]-1, tumor necrosis factor [TNF]-α, and resistin) in plasma in a subset of 382 patients. Of the 778 patients in VASST, 730 patients who had body weight and height measurements were analyzed. Patients with BMI <25 kg/m^2 ^(*n *= 276) were grouped as a reference and compared to 'overweight' (25< BMI <30 kg/m^2^, *n *= 209) and 'obese' (BMI >30 kg/m^2, ^*n *= 245) patients.

**Results:**

Obese patients had the lowest 28-day mortality followed by overweight patients while patients with BMI <25 kg/m^2 ^had the highest mortality (p = 0.02). Compared to the patients with BMI <25 kg/m^2^, obese and overweight patients also had a different pattern of infection with less lung (obese 35%, overweight 45%, BMI<25 kg/m^2 ^50%, p = 0.003) and fungal infection (obese 8.2%, overweight 11%, and BMI<25 kg/m^2 ^15.6%, p = 0.03). Per kilogram, obese and overweight patients received less fluid during the first four days (p<0.05) and received less norepinephrine (obese 0.14, overweight 0.21, BMI <25 kg/m^2 ^0.26 µg/kg/min, p<0.0001) and vasopressin (obese 0.28, overweight 0.36, BMI <25 kg/m^2 ^0.43 µU/kg/min, p<0.0001) on day 1 compared to patients with BMI <25 kg/m^2^. Obese and overweight patients also had a lower plasma IL-6 concentration at baseline (obese 106 [IQR 34-686], overweight 190 [IQR 44-2339], BMI <25 kg/m^2 ^235 [IQR 44-1793] pg/mL, p = 0.046).

**Conclusions:**

Overall obesity was associated with improved survival in septic shock and differences in pattern of infection, fluids, and vasopressors. Importantly, the magnitude of inflammatory IL-6 response is muted in the obese.

## Introduction

Obesity is a major health problem and is the sixth most important risk factor contributing to overall burden of disease worldwide [1]. Acute infection coupled with the immune response (sepsis) has been linked in multiple ways to obesity [2,3]. Obese patients were over-represented among those with serious lung injury requiring life support during the H1N1 viral pandemic [3,4], and the risk of wound infections is increased in the obese following major surgery [5]. It has also been suggested that obesity itself is a chronic inflammatory condition with increased circulating pro-inflammatory cytokines at rest [6,7]. Despite the apparent risk factors associated with obesity, it appears that in small case series obese critically ill patients, the majority of whom have severe infection, may fare better than those of normal weight [8,9]. Whether there is a true relationship between obesity and sepsis-induced morbidity and mortality remains unclear [10,11].

We conducted a retrospective analysis of the Vasopressin and Septic Shock Trial (VASST) [12] to help determine whether being overweight or obese altered (a) mortality and organ dysfunction outcomes of sepsis, (b) pattern of susceptibility to infection, (c) treatment received by patients, or (d) the inflammatory response to sepsis. To explore an inflammatory response of obese and overweight, compared with lean patients, during the initial phase of septic shock, it is well known that adipose tissues produce and release a number of pro-inflammatory mediators, including cytokines and chemokines, such as interleukin-6 (IL-6) [6,13], monocyte chemotactic protein-1 (MCP-1) [14], and tumor necrosis factor-alpha (TNF-α) [15], as well as the adipokine resistin [16]. Therefore, we assayed these cytokines at study enrollment in convenience samples of patients with septic shock in our cohort.

## Materials and methods

### Patients

We conducted a retrospective analysis in patients enrolled in a cohort of septic shock, the VASST. The VASST is a multicenter, randomized, double-blind, controlled trial evaluating the efficacy of vasopressin versus norepinephrine on mortality in 778 patients with septic shock [12]. Septic shock was defined by the presence of two or more diagnostic criteria for systemic inflammatory response syndrome (SIRS), proven or suspected infection, at least one new organ dysfunction by Brussels criteria, and hypotension despite adequate fluid resuscitation [17,18]. Of these, 730 patients had body weight and height measured at the time of enrollment of the VASST (typically about 11 hours after they first developed septic shock), and body mass index (BMI) was calculated as weight (in kilograms) divided by height (in meters) squared. Since our cohort had a small number of underweight (BMI of less than 18.5 kg/m^2^, *n *= 26) patients and these patients had similar outcomes in crude analysis compared with patients classified as normal BMI (18.5 to 24.9 kg/m^2^, *n *= 250), we used a modified form of the BMI categories endorsed by the National Institutes of Health [19], in which subjects with a BMI of less than 25 kg/m^2 ^were grouped together (*n *= 276) and were compared with 209 patients classified as overweight (BMI 25 to 29.9 kg/m^2^) and 245 patients classified as obese (BMI of at least 30 kg/m^2^). The VASST protocol was approved by the research ethics boards of all participating centers, and written informed consent was obtained from all patients or their relatives. The institutional review board for the VASST is the University of British Columbia-Providence Health Care (UBC-PHC) Research Ethics Board, and the approval number is H06-50080.

### Baseline clinical phenotype measurements

Baseline characteristics of the patients were measured at study enrollment and included age, gender, and pre-existing conditions identified on the basis of patients' medical history. Hemodynamic variables (mean arterial pressure, central venous pressure (CVP), and heart rate), tidal volume (in patients who received mechanical ventilation at study enrollment; BMI of less than 25 kg/m^2 ^*n *= 172, overweight *n *= 143, and obese *n *= 160), and baseline laboratory variables (lactate, creatinine, white blood cell count, and platelet count) were also measured.

### Outcome measurements

Our primary outcome measurement was 28-day mortality. Secondary outcomes were organ dysfunctions as previously described [20]. To assess organ dysfunction and to correct organ dysfunction scoring for deaths in the 28-day observation period, we calculated days alive and free of organ dysfunction. During each 24-hour period (8 a.m. to 8 a.m.) for each variable, days alive and free was scored as 1 if the patient was alive and free of organ dysfunction (normal or mild dysfunction using the Brussels criteria [18]). Days alive and free was scored as 0 if the patient had organ dysfunction (moderate or worse) or was not alive. Every day over the 28-day observation after intensive care unit (ICU) admission was scored in this way. Thus, the lowest score possible for each variable was 0 and the highest score possible was 28. A low score indicates more organ dysfunction, whereas a high score indicates less organ dysfunction.

### Pattern of infection

Infection was defined as culture positivity that was judged by the attending clinician of each study site to be an infection rather than just colonization. Pathogens were identified by the culture from the primary site of infection.

### Treatment

The amount of infused fluid is associated with hospital outcome [21,22]; therefore, we also tested for differences in the amount of fluid intake, output, and accumulated net fluid balance during the first four days after ICU admission (or study enrollment). We also tested for differences in infusion rates of vasopressor medications, which, in this study, were primarily norepinephrine and vasopressin. We measured serum vasopressin levels in the overweight and obese patients receiving vasopressin infusion and compared that vasopressin level with that of patients with a BMI of less than 25 kg/m^2^. Of 396 patients in the vasopressin treatment group, 54 patients had measurement of serum vasopressin level over time (baseline and 6, 24, and 72 hours) and received vasopressin infusion. Of these, 46 patients had BMI measurements and were classified as having a BMI of less than 25 kg/m^2 ^(*n *= 17) or being overweight (*n *= 13) or obese (*n *= 16) and were included in this analysis. The protocol for serum vasopressin level measurement was previously described [12].

### Inflammatory cytokine response

We assayed cytokines that have also been related to adipose tissue, including IL-6 [12], MCP-1 [13], TNF-α [14], and resistin [15], in 138 patients with a BMI of less than 25 kg/m^2^, 112 overweight patients, and 132 obese patients at enrollment. Briefly, whole-blood samples were drawn into chilled 7-mL EDTA Vacutainer tubes (BD, Mississauga, ON, Canada), put on ice immediately, and spun at 3,000 revolutions per minute for 15 minutes and then plasma was collected and stored at −70°C until further use [12]. Human 39-plex kits (EMD Millipore, Billerica, MA, USA) were used in accordance with the recommendations of the manufacturer with modifications as described below. Briefly, samples were mixed with antibody-linked magnetic beads on a 96-well plate and incubated overnight at 4°C with shaking. Plates were washed twice with wash buffer in a BioTek ELx405 washer (BioTek, Winooski, VT, USA). After 1-hour incubation at room temperature with biotinylated detection antibody, streptavidin-phycoerythrin was added for 30 minutes with shaking. Plates were washed as above, and phosphate-buffered saline was added to wells for reading by using a Luminex 200 (Illumina Inc., San Diego, CA, USA) with a lower bound of 100 beads per sample per cytokine. Each sample was measured in duplicate.

### Statistical analysis

We tested for the differences in baseline characteristics between the three groups of patients (obese, overweight, and patients with a BMI of less than 25 kg/m^2^) by using a Kruskal-Wallis test for continuous data or a chi-square test for categorical data, and we report the median and interquartile range (IQR). We evaluated the primary outcome variable (28-day mortality) by using log-rank (Mantel-Cox) test to compare Kaplan-Meier curves for patients with a BMI of less than 25 kg/m^2^, overweight patients, and obese patients. The risk of 28-day hospital mortality was expressed as hazard ratio (HR) with 95% confidence interval (CI). We tested for the influence of covariates, including APACHE II (Acute Physiology and Chronic Health Evaluation II) score, gender, diabetes history, lung infection, fungal infection, and BMI by using Cox regression analysis. Secondary clinical outcomes were days alive and free of organ dysfunction (including cardiovascular, respiratory, renal, hepatic, neurologic, and coagulation), days alive and free of SIRS (two of four SIRS criteria), and days alive and free of artificial organ support (including mechanical ventilation and hemodialysis). We tested for differences in the site of infection and the frequency of Gram-positive, Gram-negative, or fungal infection by using a chi-square test. We tested for differences in fluid amount and vasopressor administration dosages by using a Kruskal-Wallis test. We tested for differences in the vasopressin concentration over time by using one-way repeated-measures analysis of variance, and we report the mean and standard error of the mean. Serum cytokine concentrations of three patient groups were analyzed by using a Kruskal-Wallis test, and we report the median and IQR. Differences were considered significant by using a two-tailed *P *value of less than 0.05. Analyses were performed by using SPSS (version 17.0; IBM Corporation, Armonk, NY, USA) statistical software packages.

## Results

### Baseline characteristics

The median BMIs of the patients with a BMI of less than 25 kg/m^2^, overweight patients, and obese groups were 23, 28, and 34 kg/m^2^, respectively. At initial presentation, the obese group - compared with the other two patient groups - least frequently had male patients (obese 55.5%, overweight 67.9%, BMI of less than 25 kg/m^2 ^62%; *P *= 0.03), most frequently had diabetes (obese 29.8%, overweight 20.6%, BMI of less than 25 kg/m^2 ^14.9%; *P *<0.0001), had the highest CVP value (obese 15 mm Hg, overweight 14 mm Hg, BMI of less than 25 kg/m^2 ^14 mm Hg; *P *= 0.002), had the highest serum creatinine concentration (obese 191 µmol/L, overweight 150 µmol/L, BMI of less than 25 kg/m^2 ^130 µmol/L; *P *<0.0001), and was mechanically ventilated with a highest tidal volume per kilogram of predicted body weight [23] (obese 9.3 mL/kg, overweight 8.8 mL/kg, BMI of less than 25 kg/m^2 ^8.3 mL/kg; *P *<0.0001) on enrollment. There were no differences in age, APACHE II severity score, or other laboratory variables and calculations at baseline, including serum lactate and glomerular filtration rate, across the three patient groups (Table 1).

**Table 1:** Baseline characteristics compared on the basis of actual body mass index category in three groups of patients with septic shock

Baseline characteristics	Actual body mass index^a ^category, kg/m^2^			*P *value
		
	BMI <25 (*n *= 276)	BMI 25 to <30 (*n *= 209)	BMI ≥30 (*n *= 245)	
Demographics				

Age, years	63 (48-74)	64 (50-73)	63 (53-72)	0.62

Gender: Male, number (percentage)	171 (62)	142 (67.9)	136 (55.5)	0.03^b^

Race: Caucasian, number (percentage)	231 (83.7)	177 (84.7)	209 (85.3)	0.88

Body mass index^a^, kg/m^2^	23 (20-24)	28 (26-29)	34 (32-39)	<0.0001^b^

Severity of illness				

APACHE II score	27 (21-32)	26 (22-31)	27 (23-32)	0.51

Surgical condition, number (percentage)	52 (18.8)	45 (21.5)	58 (23.7)	0.4

In-hospital steroid treatment, number (percentage)	211 (76.7)	167 (79.9)	172 (70.8)	0.07

Pre-existing conditions, number (percentage)				

Chronic obstructive pulmonary disease	44 (15.9)	31 (14.8)	41 (16.7)	0.86

Congestive heart failure	19 (6.9)	14 (6.7)	22 (9.0)	0.57

Chronic liver disease	27 (9.8)	30 (14.4)	26 (10.6)	0.26

Chronic renal failure	29 (10.5)	24 (11.5)	27 (11)	0.94

Diabetes	41 (14.9)	43 (20.6)	73 (29.8)	<0.0001^b^

Ischemic heart disease	40 (15.4)	38 (20)	50 (21.8)	0.18

Chronic steroid use	67 (24.3)	42 (20.1)	51 (20.8)	0.48

Hemodynamic variables at day 1				

Central venous pressure, mm Hg	14 (10-17)	14 (11-18)	15 (12-18)	0.002^b^

Mean arterial pressure, mm Hg	55 (50-60)	56 (51-61)	55 (49-61)	0.09

Heart rate, beats per minute	130 (115-144)	125 (112-140)	125 (106-140)	0.05

Mechanical ventilation variables at day 1				

Tidal volume, mL	530 (450-630)	570 (480-650)	571 (490-650)	0.03^b^

Tidal volume, mL/kg of PBW^c^	8.3 (7.0-9.7)	8.8 (7.4-10.3)	9.3 (7.9-10.8)	<0.0001^b^

Laboratory variables at day 1				

White blood cells, x10^3^/mm^3^	14.5 (6.7-21.8)	13.1 (8.0-19.2)	12.8 (7.4-20.2)	0.72

Platelets, x10^3^/mm^3^	145 (55-256)	144 (72-232)	165 (90.5-254.5)	0.13

Creatinine, µmol/L	130 (80-218)	150 (104-266)	191 (120-297)	<0.0001^b^

GFR, mL/minute	44 (26-76)	45 (25-72)	47 (29-77)	0.27

Lactate, mmol/L	2.0 (1.2-4.2)	2.1 (0.9-3.8)	1.7 (1.1-3.5)	0.27

PaO_2_/FiO_2_	180 (120-255)	182 (123-255)	190 (135-259)	0.41

Data are presented as median (interquartile range) for continuous variables. *P *values were calculated for statistical difference between three patient groups with the use of chi-square test and Kruskal-Wallis test. ^a^Body mass index (BMI) was measured at enrollment of the Vasopressin and Septic Shock Trial (average about 11 hours after onset of septic shock). ^b^*P *<0.05. ^c^Predicted body weight (PBW) (male: 50 + 0.91 (centimeters of height: 152.4), female: 45.5 + 0.91 (centimeters of height: 152.4)). APACHE II, Acute Physiology and Chronic Health Evaluation II; GFR, glomerular filtration rate (Cockcroft-Gault formula); PaO_2_/FiO_2_, arterial oxygen partial pressure per fraction of inspired oxygen.

### Obese and overweight patients with septic shock have lower 28-day mortality and less organ dysfunction than patients with a BMI of less than 25 kg/m^2 ^despite similar severity at presentation

**Figure 1:**
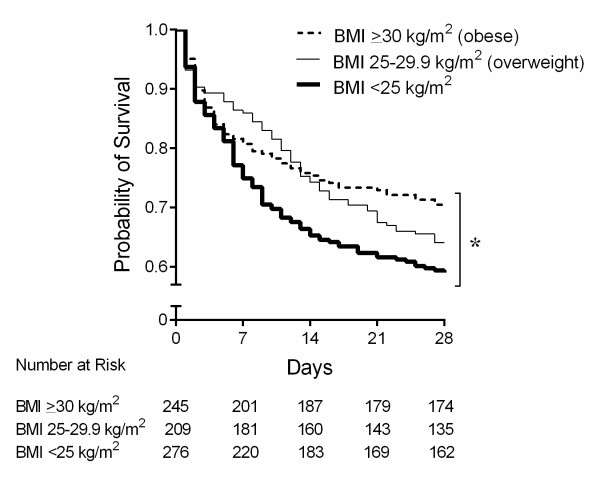
**Kaplan-Meier curves of 28-day survival compared by actual body mass index (BMI) across three groups of patients with septic shock**. Obese patients with septic shock had a significantly lowest 28-day mortality following overweight patients, whereas the septic shock patients with a BMI of less than 25 kg/m^2 ^had a highest 28-day mortality (*P *= 0.02, log-rank analysis). *P <0.05, compared among three groups.

We found that the obese and overweight patients had significantly lower 28-day mortality compared with those with a BMI of less than 25 kg/m^2 ^(*P *= 0.02) (BMI of less than 25 kg/m^2 ^versus overweight, *P *= 0.10; BMI of less than 25 kg/m^2 ^versus obese, *P *= 0.01; overweight versus obese, *P *= 0.2) (Figure 1). We introduced BMI as a continuous variable in our regression model to validate our hypothesis, following adjustment for factors that are known to influence mortality (APACHE II score) and that differ between groups at enrollment (gender, pre-existing diabetes, lung infection and fungal infection). We found that for every 1-unit increase in BMI, the HR-adjusted mortality was 2% lower (95% CI 0.97 to 0.99, *P *= 0.04) (Table 2). We also found that obese and overweight patients had significantly lower coagulation dysfunction (obese 28, overweight 23, and BMI of less than 25 kg/m^2 ^21 days alive and free of dysfunction; *P *<0.0001) and SIRS (obese 8, overweight 7, and BMI of less than 25 kg/m^2 ^4 days alive and free of two of four SIRS criteria; *P *= 0.02) compared with those with a BMI of less than 25 kg/m^2^. Differences in other organ dysfunctions were not significantly different across the three patient groups. There were no differences in days alive and free of mechanical ventilation (obese 11, overweight 10, BMI of less than 25 kg/m^2 ^6 days; *P *= 0.36) and days alive and free of renal replacement therapy (obese 25, overweight 24, BMI of less than 25 kg/m^2 ^24 days; *P *= 0.93) among the three patient groups.

**Table 2:** Hazard ratio of 28-day mortality in patients with septic shock

	Hazard ratio	95% confidence interval	*P *value
APACHE II score (per score)	1.06	1.05-1.08	<0.0001^a^

Male	1.12	0.87-1.43	0.39

Diabetes	1.05	0.78-1.41	0.77

Lung infection	1.02	0.79-1.33	0.86

Fungal infection	1.26	0.87-1.82	0.22

BMI (per score)	0.98	0.97-0.99	0.041^a^

*P *values were calculated by using Cox regression analysis. ^a^*P *<0.05. APACHE II, Acute Physiology and Chronic Health Evaluation II; BMI, body mass index.

### Pattern of infection

Compared with patients with a BMI of less than 25 kg/m^2^, obese and overweight patients had a significantly lower rate of lung infection (obese 35%, overweight 45%, and BMI of less than 25 kg/m^2 ^49.8%; *P *= 0.003) as the source of their severe sepsis and had significantly fewer fungal infections (obese 8.2%, overweight 11%, and BMI of less than 25 kg/m^2 ^15.6%; *P *= 0.03) (Table 3).

**Table 3:** Pattern of infection of patients with septic shock

	Actual body mass index category, kg/m^2^			*P *value
		
	BMI <25 (*n *= 276)	BMI 25 to <30 (*n *= 209)	BMI ≥30 (*n *= 245)	
Primary source of infection, number (percentage)				

Lung	137 (49.8)	94 (45)	85 (35)	0.003^a^

Abdomen	69 (25.1)	54 (25.8)	73 (30)	0.41

Genitourinary	10 (3.6)	11 (5.3)	13 (5.3)	0.58

Blood	13 (4.7)	14 (6.7)	16 (6.6)	0.57

Skin	17 (6.2)	14 (6.7)	26 (10.7)	0.12

Other^b^	23 (8.4)	21 (10)	25 (10.3)	0.72

Pathogen type in cultures, number (percentage)				

Gram-positive	81 (29.5)	53 (25.4)	81 (33.3)	0.18

Gram-negative	68 (24.7)	52 (24.9)	46 (18.9)	0.21

Fungal	43 (15.6)	23 (11)	20 (8.2)	0.03^a^

^a^*P *<0.05. ^b^Bone/joint/central nervous system infection. *P *values were calculated for statistical difference between three patient groups with the use of chi-square test. BMI, body mass index.

### Obese and overweight patients receive less intravenous fluids and vasopressors than patients with a BMI of less than 25 kg/m^2^

**Figure 2:**
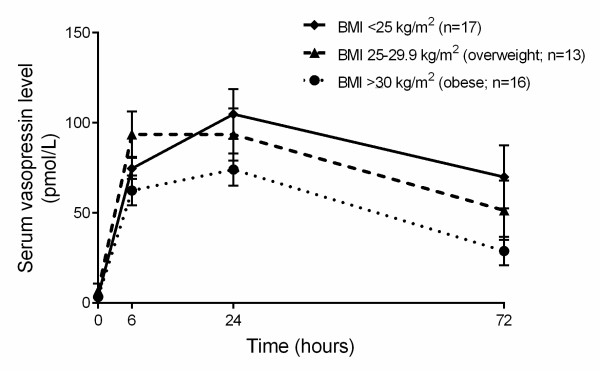
**Serum vasopressin level during septic shock compared across the three patient groups**. At 24 and 72 hours after vasopressin infusion, the overweight and obese patients had a trend toward lower mean vasopressin concentrations compared with those with body mass index (BMI) of less than 25 kg/m^2 ^(*P *= 0.08, one-way repeated-measures analysis of variance). Error bars indicate standard error of the mean.

The updated Surviving Sepsis Campaign guidelines suggest titrating fluid therapy to body mass with an initial prescription of at least 30 mL/kg [24]. Further mass-adjusted fluid administration is prescribed as required. It is now appreciated that, whereas adequate early fluid resuscitation is crucial, overzealous fluid administration is harmful [21,22]. During the initial resuscitation for septic shock, obese and overweight patients received significantly less fluids per kilogram than those with a BMI of less than 25 kg/m^2 ^(Table 4). This appears driven by non-body weight-adjusted fluid prescription, because there was no significant difference in absolute fluid administered between groups (Table 4). Although vasopressin is traditionally prescribed without body weight adjustment, some have suggested that vasopressin be prescribed with it [25]. During the initial phase of septic shock (day 1), both norepinephrine (obese 0.14, IQR 0.09 to 0.25; overweight 0.21, IQR 0.12 to 0.34; BMI of less than 25 kg/m^2 ^0.26, IQR 0.15 to 0.44 µg/kg per minute; *P *<0.0001) and body weight-adjusted vasopressin (obese 0.28, IQR 0.23 to 0.33; overweight 0.36, IQR 0.31 to 0.40; BMI of less than 25 kg/m^2 ^0.43, IQR 0.38 to 0.50 µU/kg per minute; *P *<0.0001) dosage - similar to fluid administration - were prescribed at significantly lower doses in obese and overweight patients compared with those with a BMI of less than 25 kg/m^2^. At 72 hours of vasopressin infusion, obese and overweight patients had a trend toward lower serum vasopressin level (obese 28.9 ± 7.9, overweight 51.5 ± 16.5, BMI of less than 25 kg/m^2 ^69.9 ± 17.5 pmol/L; *P *= 0.08) compared with patients with a BMI of less than 25 kg/m^2 ^receiving vasopressin infusion (Figure 2).

**Table 4:** Fluid administration during the first four days after septic shock

	Amount of fluid, mL				Amount of fluid per body weight, mL/kg			
	**BMI <25** **(*n *= 276)**	**BMI 25 to <30** **(*n *= 209)**	**BMI ≥30** **(*n *= 245)**	***P *value**	**BMI <25** **(*n *= 276)**	**BMI 25 to <30** **(*n *= 209)**	**BMI ≥30** **(*n *= 245)**	***P *value**

Predicted body weight^a^, kg	67 ± 2.1	64 ± 0.7	61 ± 1.0	0.003^b^	-	-	-	

Day 1								

Intake	11,000 ± 310	10,500 ± 360	10,500 ± 310	0.27	180 ± 5	130 ± 5	100 ± 3	<0.0001^b^

Output	4,100 ± 200	3,900 ± 240	3,900 ± 190	0.7	60 ± 3	50 ± 3	40 ± 2	<0.0001^b^

Net accumulated balance	7,000 ± 330	6,600 ± 360	6,700 ± 330	0.43	110 ± 5	84 ± 5	70 ± 3	<0.0001^b^

Day 2								

Intake	16,000 ± 420	15,200 ± 500	15,400 ± 420	0.28	250 ± 7	190 ± 6	150 ± 5	<0.0001^b^

Output	6,500 ± 270	6,400 ± 320	6,400 ± 290	0.95	100 ± 5	80 ± 4	60 ± 3	<0.0001^b^

Net accumulated balance	9,500 ± 430	8,800 ± 490	9,000 ± 460	0.28	150 ± 7	110 ± 6	90 ± 5	<0.0001^b^

Day 3								

Intake	19,900 ± 520	19,000 ± 600	19,400 ± 510	0.37	320 ± 9	240 ± 8	190 ± 5	<0.0001^b^

Output	9,100 ± 360	8,900 ± 410	9,300 ± 410	0.95	140 ± 6	110 ± 5	90 ± 4	<0.0001^b^

Net accumulated balance	10,800 ± 490	10,200 ± 580	10,200 ± 540	0.31	170 ± 8	130 ± 7	100 ± 5	<0.0001^b^

Day 4								

Intake	23,600 ± 590	23,000 ± 710	23,300 ± 590	0.41	380 ± 10	290 ± 9	230 ± 7	<0.0001^b^

Output	12,000 ± 430	12,300 ± 530	12,600 ± 530	0.9	190 ± 7	160 ± 7	120 ± 6	<0.0001^b^

Net accumulated balance	11,700 ± 540	10,700 ± 660	10,700 ± 600	0.17	190 ± 9	140 ± 8	110 ± 6	<0.0001^b^

Data are presented as mean ± standard error of the mean. *P *values were calculated for statistical difference between three patient groups with the use of Kruskal-Wallis test. ^a^Predicted body weight (male: 50 + 0.91 (centimeters of height: 152.4), female: 45.5 + 0.91 (centimeters of height: 152.4)). ^b^*P *<0.05. BMI, body mass index.

### IL-6 levels of obese and overweight patients are muted compared with those of patients with a BMI of less than 25 kg/m^2^

There was no significant difference in body temperature (obese 38.6, IQR 37.8 to 39.4; overweight 38.5, IQR 37.8 to 39.1; BMI of less than 25 kg/m^2 ^38.4, IQR 37.5 to 39.1°C; *P *= 0.053), platelet count (obese 165,000, IQR 90,500 to 254,500; overweight 144,000, IQR 72,000 to 232,000; BMI of less than 25 kg/m^2 ^145,000, IQR 55,000 to 256,000/mm^3^; *P *= 0.13), and white blood cell count (obese 12,800, IQR 7,400 to 20,200; overweight 13,100, IQR 8,000 to 19,200; BMI of less than 25 kg/m^2 ^14,500, IQR 6,700 to 21,800/mm^3^; *P *= 0.72) between groups.

**Figure 3:**
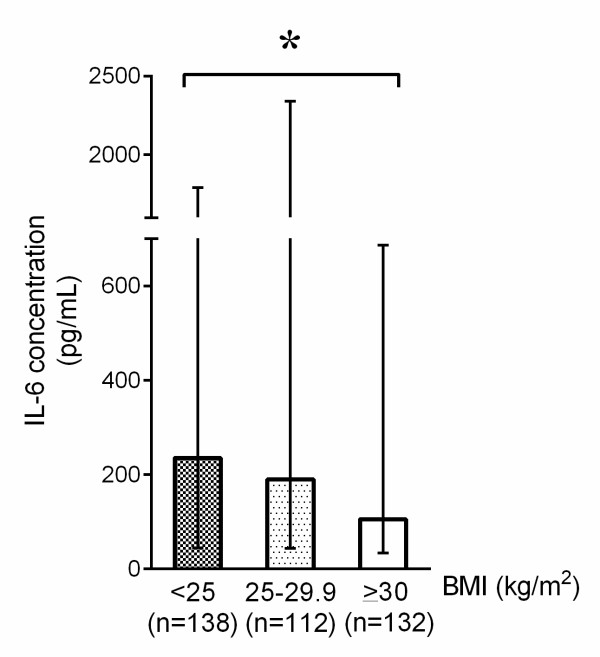
**Plasma interleukin-6 (IL-6) expression differences at the initial phase of septic shock compared across the three patient groups**. Compared with patients with a body mass index (BMI) of less than 25 kg/m^2^, overweight and obese patients had significantly lower IL-6 (*P *= 0.046) plasma levels at the initial phase of septic shock. *P *values were calculated by using the Kruskal-Wallis test. Error bars indicate interquartile range. *P <0.05.

We found that, in the early phase of septic shock, plasma IL-6 (obese 106, IQR 34 to 686; overweight 190, IQR 44 to 2,339; BMI of less than 25 kg/m^2 ^235, IQR 44 to 1,793 pg/mL; *P *= 0.046) levels were significantly lower in obese and overweight patients than in those with a BMI of less than 25 kg/m^2 ^(Figure 3). There was no difference in plasma MCP-1 (obese 567, IQR 220 to 1,817; overweight 842, IQR 284 to 2,386; BMI of less than 25 kg/m^2 ^782, IQR 315 to 2,686 pg/mL; *P *= 0.06), resistin (obese 77, IQR 39 to 137; overweight 66, IQR 37 to 134; BMI of less than 25 kg/m^2 ^73, IQR 38 to 134 ng/mL; *P *= 0.85), or plasma TNF-α in obese or overweight patients versus those with a BMI of less than 25 kg/m^2 ^(obese 15, IQR 6 to 29; overweight 14, IQR 7 to 31; BMI of less than 25 kg/m^2 ^13, IQR 6 to 33 pg/mL; *P *= 0.86).

## Discussion

Our major finding was that despite equal severity of illness upon presentation, mortality in obese and overweight patients was significantly lower than in patients with a BMI of less than 25 kg/m^2 ^(Figure 1). Compared with those with a BMI of less than 25 kg/m^2^, obese and overweight patients had less frequent lung and fungal infections as the site and organism causing septic shock. We also found that obese and overweight patients were treated differently, in what appeared to be a 'one size fits all' non-weight-adjusted dosing, so that obese and overweight patients received less fluids and vasopressors per kilogram (norepinephrine and vasopressin) than patients with a BMI of less than 25 kg/m^2^. Unlike previous reports of obesity augmenting the inflammatory response [6,7,13], we found that the IL-6 inflammatory response was muted in overweight and obese patients compared with those with a BMI of less than 25 kg/m^2 ^in early septic shock. This surprising result is novel and consistent with current and previous reports of improved survival outcomes in obese patients [8,9].

Conceivably, the inclusion of very underweight or very overweight patients could alter this analysis and interpretation. We removed the underweight group (*n *= 26) and re-analyzed for mortality difference and found the same significant result in which obese patients had the lowest 28-day mortality followed by overweight and normal-BMI patients. A similar *post hoc *analysis of the morbidly obese (BMI ≥40 kg/m^2^) showed that this group had a lower 28-day mortality than all other weight groups, consistent with a previous report by Abhyankar and colleagues [26]. We also found that despite a higher prevalence of diabetes, those with obesity had a decreased risk of hospital mortality. Covariate adjustment of diabetes appeared to augment this protection for the obese. We found that BMI was an independent predictor of lower mortality with an HR of 0.98 per kg/m^2 ^(95% CI 0.97 to 0.99, *P *= 0.041), suggesting that although diabetics had a poorer prognosis, diabetes itself had a weaker association with mortality than BMI (Table 2). Our results are in agreement with data from the Acute Decompensated Heart Failure National Registry, which studied patients with acute heart failure and found that hospital mortality rates decreased in a near-linear fashion across higher BMI. For every 5-unit increase in BMI, the odds of risk-adjusted mortality were 10% lower (95% CI 0.88 to 0.93, *P *<0.0001) [10]. Our findings also align with a meta-analysis in critically ill patients which demonstrated that patients who were obese (had a BMI of 30 to 39.9 kg/m^2^) had significantly lower mortality compared with the non-obese patients (BMI of less than 30 kg/m^2^) (relative risk 0.86, 95% CI 0.81 to 0.91; *P *<0.001) [11].

Previous studies demonstrate that low tidal volume ventilation is associated with a lower mortality [23], which might have contributed to our findings. However, we found the opposite. Obese and overweight patients received higher tidal volume ventilation compared with patients with a BMI of less than 25 kg/m^2^, particularly when corrected to predicted body weight (Table 1).

Although our data do not allow us to determine whether there was an absolute difference in susceptibility to infection (since we would need to know the number of obese and non-obese patients at risk), we did observe a substantial difference in pattern of infection. We observed a large and significant reduction in the fraction of patients having lung infections and the fraction of patients having fungal infections in obese patients. One possible limitation to this finding is that, in this patient cohort, the diagnosis of lung infection was based primarily on physician judgment, which depended on results from chest radiographs and sputum culture. This could have been confounded by the increasing inability to obtain clear or definitive chest radiographs in obese patients. Although there was no statistically significant difference in white blood cell counts or a baseline difference in immunosuppression or chronic steroid use, we did find that obese and overweight patients expressed significantly lower plasma IL-6 levels during the initial phase of septic shock compared with those with a BMI of less than 25 kg/m^2^. This is in agreement with a study of patients with blunt injury which found that obese patients had a significantly lower inflammatory cytokine profile than those with a normal BMI [27]. Why obese patients have an altered IL-6 inflammatory response is not known. However, the finding of decreased circulating IL-6 concentrations in obese patients with sepsis is consistent with the observation of improved survival outcome. We also measured resistin, an adipokine that antagonizes the effects of insulin [16] and that functions as a pro-inflammatory cytokine [28]. Unlike in stable obese patients in whom resistin levels are much higher than those with a normal BMI, we found no significant difference between obese and normal-BMI patients in early septic shock.

Unlike in most medical therapies which rely upon weight-based dosing, we hypothesized that the empirical manner (1 L of fluid at a time mentality) in which fluid and vasopressor therapy are recommended for sepsis [22,24] would cause obese and overweight patients to receive relatively less weight-adjusted fluids and vasopressors than normal-BMI patients. Alternatively, the treating physicians may have recognized that blood volume is not linearly related to weight [29,30] and, therefore, may have followed the current Surviving Sepsis Campaign guidelines that suggest that fluid administration be based on ideal body weight, which did not differ between the three groups in this study (Table 4). Indeed, obese and overweight patients received less body weight-adjusted fluids, norepinephrine, and vasopressin in early septic shock compared with the patients with a BMI of less than 25 kg/m^2^. As excessive fluid and vasopressor resuscitation is associated with increased mortality [21,22], it may be that using doses more typically used for smaller individuals was, ironically, protective. To directly assess whether this was the case, we measured vasopressin levels in patients randomly assigned to receive this drug in the protocolized non-body weight-adjusted dose used in the VASST. We found that, compared with those with a BMI of less than 25 kg/m^2^, obese and overweight patients had a trend toward lower serum vasopressin concentrations after 24 hours of vasopressin infusion. The net accumulated fluid balance differs by only 1 L between patients of normal weight and obese patients. It could be argued that this small difference is unlikely to account for the observed outcome differences. However, indexed to body mass, this amounts to a difference of 80 mL/kg, a remarkable difference that conceivably could be related to outcomes. In addition, although there was a statistically significant difference in CVP values between the three patient groups, which was possibly due to differences in chest wall compliance in the obese compared with the normal-BMI patients, the clinical significance of this small difference is uncertain.

The strengths of this study are, first, that our cohort is a large prospective multicenter cohort of patients with well-defined septic shock [12]. Second, our results after covariate-adjusted and unadjusted analyses were consistent.

There are several important limitations to our study. APACHE II score has never been validated in obese patients. For example, oxygenation may be worsening despite normal lung function or creatinine level may be increased despite normal kidney function. Thus, obese patients may have artificially elevated APACHE II scores. However, there was no difference of serum lactate levels between the three patient groups at enrollment. Because our analysis was retrospective, some additional useful data such as coexisting hypertension, serum cholesterol, or other serum adipokines such as leptin and adiponectin which regulate insulin sensitivity were not recorded in the dataset. In addition, adipose tissue-related cytokines in our analysis are released not only from adipose tissue but also from other tissues and this could affect our interpretation. Also, our study may represent not the 'real' population of obese patients with sepsis but only an obese population included in a randomized controlled study with a strict inclusion criteria.

## Conclusions

We demonstrated that, in a cohort of patients with septic shock, the obese and overweight patients had decreased mortality compared with patients with a BMI of less than 25 kg/m^2^. Obese and overweight patients may have had less aggressive disease, as shown by our evaluations of blunted host inflammatory response to pathogens (lower IL-6), and an altered pattern of infection (less frequent lung and fungal infection). Obese and overweight patients may also have been protected by receiving less fluids and vasopressors compared with the patients with a BMI of less than 25 kg/m^2^.

## Key messages

• In septic shock, the obese and overweight patients had decreased mortality compared with patients with a BMI of less than 25 kg/m^2^.

• Compared with patients with a BMI of less than 25 kg/m^2^, obese and overweight patients with sepsis had a lower rate of lung infection as the source of their severe sepsis as well as fewer fungal infections.

• In septic shock, the obese and overweight patients receive less intravenous fluids and vasopressors per kilogram than patients with a BMI of less than 25 kg/m^2^.

• At the initial phase of septic shock, plasma IL-6 concentrations in obese and overweight patients are muted compared with those of patients with a BMI of less than 25 kg/m^2^.

## Abbreviations

APACHE II: Acute Physiology and Chronic Health Evaluation II; BMI: body mass index; CI: confidence interval; CVP: central venous pressure; HR: hazard ratio; ICU: intensive care unit; IL-6: interleukin-6; IQR: interquartile range; MCP-1: monocyte chemotactic protein-1; SIRS: systemic inflammatory response syndrome; TNF-α: tumor necrosis factor-alpha; VASST: Vasopressin and Septic Shock Trial.

## Competing interests

The authors declare that they have no competing interests.

## Authors' contributions

PW and KRW contributed to study conception and design, acquisition of data, statistical analysis, interpretation of data, and drafting of the manuscript. JAR and JHB contributed to study conception and design, acquisition of data, interpretation of data, and drafting of the manuscript. All authors read and approved the final manuscript.
